# Health Effects of Coastal Storms and Flooding in Urban Areas: A Review and Vulnerability Assessment

**DOI:** 10.1155/2013/913064

**Published:** 2013-05-30

**Authors:** Kathryn Lane, Kizzy Charles-Guzman, Katherine Wheeler, Zaynah Abid, Nathan Graber, Thomas Matte

**Affiliations:** ^1^Bureau of Environmental Surveillance and Policy, New York City Department of Health and Mental Hygiene, New York, NY 10013, USA; ^2^Bureau of Environmental Disease Prevention, New York City Department of Health and Mental Hygiene, New York, NY 10013, USA

## Abstract

Coastal storms can take a devastating toll on the public's health. Urban areas like New York City (NYC) may be particularly at risk, given their dense population, reliance on transportation, energy infrastructure that is vulnerable to flood damage, and high-rise residential housing, which may be hard-hit by power and utility outages. Climate change will exacerbate these risks in the coming decades. Sea levels are rising due to global warming, which will intensify storm surge. These projections make preparing for the health impacts of storms even more important. We conducted a broad review of the health impacts of US coastal storms to inform climate adaptation planning efforts, with a focus on outcomes relevant to NYC and urban coastal areas, and incorporated some lessons learned from recent experience with Superstorm Sandy. Based on the literature, indicators of health vulnerability were selected and mapped within NYC neighborhoods. Preparing for the broad range of anticipated effects of coastal storms and floods may help reduce the public health burden from these events.

## 1. Introduction

With its densely populated and highly developed coastline, New York City faces significant risks from flooding, especially during coastal storms. Due to sea level rise caused by climate change, flooding associated with coastal storms and hurricanes is expected to increase in intensity, frequency, and duration. Intense hurricanes may also become more frequent [1]. In October 2012, Superstorm Sandy brought these vulnerabilities into stark relief, causing a record storm surge, extensive flooding, loss of life, injury, widespread power outages, and widespread damage to property and coastal neighborhoods in Queens, Manhattan, Staten Island, Brooklyn, and throughout the region. As of November 26, 2012, NYC had estimated that public and private losses in the city totaled at least $19 billion [2].

Many people living near the coasts may be vulnerable. From 1970 to 2010, the population in coastal US areas has increased by 39%, and population density along the coasts is expected to continue to increase [3]. Many residents are older adults, a group that is particularly vulnerable to the effects of storms and flooding [4]. People living in coastal areas will need to prepare for a wide variety of potential health impacts [5].

Quantification of the future burden of health outcomes from coastal storms is difficult. Many factors can influence the health impact of storms, including the severity and other characteristics of the storm, the exact timing and location of landfall, and the unique geographic and topographic characteristics of the affected area. For instance, although Superstorm Sandy was officially classified as a post-tropical storm by NOAA when it made landfall, it had several characteristics that produced a record storm surge of over 13 feet at Battery Park in lower Manhattan—including making landfall during high tide and combining with a midlatitude trough system that increased the power and size of the storm. Local housing characteristics and infrastructure, the existence and execution of evacuation and other emergency plans, and underlying population health and resilience also affect the impacts of storms. 

Lessons learned from Sandy, Hurricane Katrina, and other devastating storms, however, shed light on what adverse outcomes are possible and what factors may make people and neighborhoods more or less vulnerable to their impacts. This information can be used for adaptation planning. Preparing for a range of anticipated health impacts of coastal storms and floods could help reduce the health burden from these events. To this end, we conducted a broad review of the health impacts of US coastal storms, with a focus on outcomes relevant to New York City and urban coastal areas. We also identified population-level indicators that may be useful in identifying vulnerable neighborhoods. Vulnerability mapping can help planners and communities better understand the baseline health status of neighborhoods in the evacuation zones and some of the factors that may make residents more vulnerable to a range of health effects during and after a major storm.

## 2. Materials and Methods

Recent literature about the health impacts of coastal storms and floods, such as injuries, depression, anxiety, and poor physical health, was reviewed. The intent was not to be exhaustive but to describe the range of potential health effects that could occur in NYC and other urban areas and describe likely and/or potentially severe outcomes. Using the US National Library of Medicine's PubMed database, we searched a set of general terms relating to storms and flooding to capture a broad range of health outcomes:

 (cyclonic storms OR floods OR hurricane) AND (mental health OR health OR injury OR morbidity) AND United States.

Approximately 300 articles were identified and 90+ abstracts were reviewed. A total of 70 published studies, which covered a wide range of potential exposures and adverse health outcomes, were compiled and reviewed in detail. Logic models were created to visualize the causal pathways through which health outcomes occur. This review was largely completed prior to Superstorm Sandy, and the lessons learned and health impacts from that incident are still under study. Nonetheless, some initial lessons learned from the response to Superstorm Sandy were qualitatively considered in this review. 

Based on vulnerable subgroups identified in the literature, potential indicators of population vulnerability for which data are available were identified and mapped within the 42 NYC United Hospital Fund (UHF) neighborhoods located within any NYC hurricane evacuation zone. UHF neighborhoods are zip code-aggregated areas within all five boroughs. For each indicator, prevalences were categorized into quartiles by neighborhood. 

## 3. Results and Discussion

**Figure 1:**
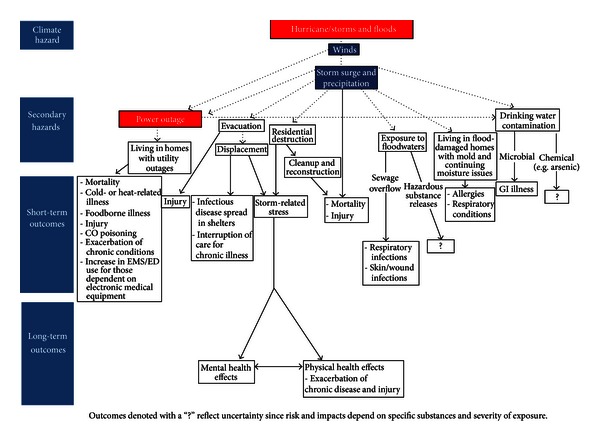
Logic model of potential health impacts of coastal storms.

Health outcomes can occur through multiple pathways (see Figure 1) including (1) hazards from exposure to storm impact; (2) evacuation; (3) post-storm hazards from utility outages and sheltering in place in inadequate housing; (4) exposure to secondary hazards including contaminated drinking water, contact with contaminated floodwaters, and mold and moisture in housing; (5) population displacement and disruption of services; (6) mental health effects from traumatic or stressful experiences during and after the storms and (7) health and safety risks from clean-up and recovery activities.

### 3.1. Hazards of Storm Exposure

The most severe acute effect of hurricane landfall is death from drowning, electrocutions, or physical trauma [6–9]. Older age increases the risk of death—nearly 85% of people killed during and in the immediate aftermath of Hurricane Katrina were aged 51 and older, and almost half were older than 75 years of age [9]. Residents of nursing homes that sheltered in place were among those killed by drowning [10]. As with other natural disasters, low-income populations may be particularly vulnerable [11]. 

The causes and age pattern of deaths during the impact phase of Superstorm Sandy were generally consistent with prior storms. In the acute phase, Sandy caused 43 deaths in NYC. Death was caused most frequently by drowning associated with the storm surge (*n* = 34, 79%). Other deaths were caused by falling trees, falls, electrocution, and other trauma. Nearly half of fatalities occurred among adults aged 65 or older (*n* = 20, 47%), and more than half of deaths occurred on Staten Island (*n* = 23, 53%). Epidemiologic studies will be needed in the coming months and years, however, to assess the storm's full impact on excess mortality from accidental and natural causes, as well as other health impacts, in impacted communities.

Heavy precipitation weather events can also cause flash flooding, which occurs when water from heavy rains collects in a relatively short time and runoff is accelerated in mountainous or narrow valley terrain [12]. Nationally, flash floods are the most common cause of flood deaths via drowning [12, 13]. Flash floods occur in NYC but historically have rarely been life threatening because of local topography. However, much of NYC's infrastructure, especially in low-lying or poor drainage areas, cannot cope with more than one inch per hour of rainfall [14]. 

Hurricane landfall can also result in a range of non-fatal injuries, including blunt trauma, puncture wounds, lacerations, sprains/strains, motor vehicle crashes, animal bites, and electrocution [15–18]. Falls, traffic accidents, and other injuries can also occur in period immediately before a major storm hits, particularly among the elderly, as people try to evacuate and prepare their residences [18]. 

Those who do not evacuate prior to a storm and shelter in place, by choice or necessity, may risk injury or death during a coastal storm [9]. Poor and minority populations, and elderly nursing home residents, are more likely to lack transportation during disasters [19]. These populations often have a high prevalence of chronic health problems, which increases their vulnerability to other storm-related hazards. 

Exposure to natural disasters may trigger acute coronary syndromes [20]. Researchers have also observed a 3-fold increase in the incidence of acute myocardial infarction among Tulane Health Sciences Center hospital patients two years after Hurricane Katrina related to emotional stress [21]. 

### 3.2. Evacuation

While evacuations of health care facilities are undertaken to avoid health risks that result from major destruction such as flooding, power outages, and interruptions in medical care if health facilities are rendered inoperable, safe evacuations require advance planning and technical expertise. In the evacuation phase, frail or medically incapacitated people may need assistance getting to trains or buses or may require other modes of transport. For municipal health and transit staff, as well as private agencies serving this population, this represents a large undertaking, requiring organization, training, and adequate staffing, which could be difficult to ensure during an emergency. Furthermore, receiving facilities located outside the storm surge zone will require additional supplies and patient care capacity. Delayed (post-storm) evacuation of health care facilities can compound logistical problems and risks because of loss of power and damage to communications and transportation infrastructure, making it more difficult to transport and track patients who may already be compromised by failure of medical equipment or exposure to heat or cold [22].

A survey of twenty Gulf state nursing homes identified significant logistical and response problems during Hurricane Katrina related to transportation, staffing, maintaining complex medication regimens, insufficient emergency provisions, and failure to follow emergency plans [23]. All facilities encountered problems, but there were slightly more negative effects (including dehydration, depression, and skin tears) reported by administrators from facilities that evacuated rather than sheltered in place. Nursing home administrators reported little support from state and local emergency responders in evacuation decisions and implementation [23].

In NYC, five hospitals shut down due to Sandy. Three of them—NYU Langone Medical Center, Bellevue Hospital, and Coney Island Hospital—had to evacuate patients after the storm hit because of flood damage to critical equipment located in low-lying parts of the hospitals. Many nursing and long-term care facilities also lost power and had to evacuate patients.

### 3.3. Secondary Hazards, Including Utility Outages and Sheltering in Place in Inadequate Housing

Local or widespread power outages could result from flood damage to underground and low-lying electrical infrastructure, as well as damage to utility poles and aboveground wires from high winds and downed trees. 

Following widespread power outages, carbon monoxide (CO) poisoning is a major health hazard. Deaths and illness can occur when portable generators, cooking appliances, and other fuel burning equipments are used indoors or improperly [24–34]. 

Mortality from other accidental and natural causes may also increase during power outages. A study of the August 14-15, 2003, Northeast blackout that affected NYC found increased mortality from both accidental and natural causes that resulted in approximately 90 excess deaths (an increase of 28%) [35]. Seniors aged 65–74 years were most vulnerable. Researchers theorized that increased mortality could be related to more physical demands on vulnerable people due to non-functioning elevators and subways, increased call times for ambulances, closed stores and pharmacies, and potentially the effects of increased exposure to air pollution and ambient air temperatures [35]. 

Power losses may lead to increased emergency medical services (EMS) calls and emergency department (ED) visits from patients who rely on electrically powered medical equipments like ventilators and oxygen [24, 27, 32, 34, 36–39]. Frail residents of nursing homes and other health care facilities that shelter in place can be especially vulnerable to power outages in facilities without backup generators or with backups located in flood-vulnerable places such as basements. Risks include the failure of medical equipment and exposure to hot or cold ambient temperatures, possibly compounded by a lack of supplies or adequate staff in facilities that are not well prepared [22, 40]. Hospital admissions for respiratory conditions may also increase [41].

Incarcerated populations are also at potentially high risk. In New Orleans during Hurricane Katrina, a lack of emergency preparedness and planning by corrections officials led to chaos during the storm at the over-crowded Orleans Parish Prison. Prisoners locked in cells were left alone without power, food, water, or even sufficient ventilation during the storm. Prisoners described lack of access to medical care and interruptions in care for serious chronic illness during and after the storm [42]. Prison populations often have higher rates of mental and physical illness and substance use disorders than the general population and are at risk for exacerbations [43].

After Superstorm Sandy made landfall, hundreds of thousands of NYC residents initially lost power. However, even after the electric grid had been largely restored, many residential buildings in storm-inundated areas still lacked electric power, heat, or running water, often because of salt water flood damage to buildings electrical and heating systems. Many people who did not evacuate in advance of the storm sheltered in place in housing conditions that lacked one or more of these essential services. Exposure to hot or cold ambient temperatures from lack of climate control could result in heat- or cold-related illness, including heat stroke or hypothermia, as well as exacerbation of respiratory, cardiovascular, and other chronic diseases [41, 44, 45].

In the days following Sandy, health department surveillance data reflected the impact of people living without power or heat and, in some cases, trying to provide power or heat in unsafe ways. From the storm impact until November 9 (10 days), CO-related emergency department visits and Poison Center (PCC) calls for CO exposure were elevated for the time of year; PCC data frequently identified storm-related sources of exposure including charcoal grills and household cooking appliances used for heating, as well as portable generators. Counts of cold illness syndrome emergency department visits (including hypothermia) were also elevated through November 9 [46].

While the potential health impacts resulting from sheltering in inadequate housing after a storm have not yet been formally evaluated, exacerbation of chronic health conditions, including physical, mental, and substance use disorders, could occur from a disruption of care due to lack of light, telecommunications, and elevator service that makes it difficult for people to access outside care, obtain medications, maintain self-care, and receive home-based care services. There may also be stress-related exacerbation of chronic physical and mental health problems related to isolation. 

Injuries could include fire risks among those using stoves for heat or candles for light [47] and risk of falls from inadequate lighting in dwellings, hallways, and stairwells [25]. Inability to access food and fresh water, particularly for people living in high-rise apartments where water delivery is dependent on electric pumps, could lead to infectious disease risk because of inability to properly wash hands or food, bathe, or flush toilets.

People living in residential buildings without electricity may be more vulnerable to foodborne disease because they cannot refrigerate food. After a 2003 summer power outage in NYC, increases in diarrheal illness caused by the consumption of spoiled foods, especially meat and seafood, were observed [48]. Furthermore, unless active steps are taken to remove spoiled food from residences and restaurants (which typically pay private contractors to collect waste) during prolonged outages, pest populations could increase [24]. It is possible that proliferation of pests, including rodents and roaches, because of difficulty with cleaning and removing trash may exacerbate allergies, asthma, and other respiratory conditions.

Older adults, young children, individuals with pre-existing health conditions, and those living on the upper floors of high-rise buildings or those who are disabled may be especially vulnerable to health effects resulting from power and utility outages. Safety concerns stemming from lighting outages and disabled safety systems in hallways and stairwells may deter this population from seeking or receiving assistance. In addition, it is possible that this population may be at risk for mental health outcomes observed in populations who have experienced long-term displacement, including post-traumatic stress and other mental health problems.

### 3.4. Secondary Hazards from Contaminated Water and Mold and Moisture in Housing

Exposure to mold in flood-damaged buildings could worsen allergic and asthmatic symptoms—among those with pre-existing allergic sensitization—and respiratory infections [49]. Although increased concentrations of outdoor and indoor mold were detected after Hurricane Katrina in areas that had experienced flooding [49, 50], the impact of the mold on health following Katrina has not been well characterized, possibly because of under-reporting or under-detection of health problems, or by population displacement that reduced exposure [49].

Coastal storms can cause release of untreated sewage through direct damage and flooding of treatment facilities, power outage, or combined sewage overflows (CSO). CSOs are caused when heavy rains overwhelm combined systems of collecting storm and precipitation water runoff causing the discharge of untreated sewage into rivers. Secondary exposure to sewage-contaminated floodwaters and wastewater, along with impaired access to potable water and flushable toilets, may lead to gastrointestinal infections, acute respiratory infections, skin infections, and insect bites [15, 16, 51, 52]. There may also be a risk of increases in vectorborne diseases like West Nile virus because sites where water collects after heavy rains could become breeding grounds for mosquitoes.

Rainfall, wind, and runoff in the watershed area can contribute to high turbidity levels, which can interfere with the disinfection process of drinking water. In NYC, a coastal storm will not necessarily impact the drinking water supply because the watershed and reservoirs are located up to 125 miles from the city. However, an increasing number of heavy precipitation events in the watershed may lead to more instances of high turbidity levels. The NYC Department of Environmental Protection monitors turbidity at over 1,400 locations in the watershed and distribution system and activates the Ashokan Waste Channel to reduce turbidity levels [53].

Storm damage may compromise sites storing toxic waste, and flood waters may move hazardous substances to new areas. Following Hurricane Katrina, hazardous substances such as volatile organic compounds (VOCs), lead, and arsenic were detected in the air, soil, and sediment samples. No health effects have been directly observed in a storm-specific context [54–59]. However, the potential for a toxic release of hazardous substances after a storm exists [55–57]. Following Sandy, initial testing of two Superfund sites indicated that contact with contaminated water from these areas was not a major health threat [60].

### 3.5. Population Displacement, Shelters, and Health Care Disruption

Displacement led to a host of adverse effects following Hurricane Katrina and major storms in other areas. Health risks were often related to living in congregate shelters, disruption of access to health care, or some combination of both. 

The spread of infectious diseases, such as norovirus, was documented among residents of temporary shelters or evacuation centers in the wake of Hurricane Katrina [61]. Waterborne illnesses and infectious disease spread in shelters have been more frequently observed among young children and infants with naïve immune systems [61, 62]. 

Displacement can lead to interruption in medical care and exacerbation of chronic health conditions. Contributing factors to disruption of care after Katrina, Hurricane Ike, and other natural disasters included evacuees without medical history information, medications or knowledge of medication names and doses, and access to medical records [63, 64]. Following Hurricane Katrina, health care providers and focus groups also reported that chronic disease treatment interruptions—especially among patients with cancer, hypertension, end stage renal disease, cardiovascular disease, and respiratory illnesses—were problems [63]. Dialysis sessions may be missed [65]. In a sample survey of Louisiana Red Cross shelter residents in the weeks immediately following Katrina, slightly more than half of the residents had chronic medical conditions including hypertension, hypercholesterolemia, diabetes, and lung diseases [64]. About a third needed acute medical attention. Shelter residents may have pre-existing mental health conditions that require ongoing care [64].

After Hurricane Katrina, there was also a need for urgent medical supplies, such as oxygen tanks [63]. Emergency responders may also need access to sufficient quantities of vaccines (and facilities with proper storage capabilities), medications, and other preventive medical supplies for displaced populations.

Women who are displaced may have trouble accessing contraceptives and other reproductive health services. Following Hurricane Ike in Texas, racial disparities in access to contraceptives were also observed [66]. Disruptions in prenatal care for pregnant women, including folate supplementation, could potentially lead to adverse birth outcomes [67, 68]. 

Displacement can also result in other physical health effects. Displacement at 12 months following Katrina was associated with increased risk of hip fracture among seniors, particularly among women, those with co-morbidities and a history of hip fracture [69]. Among nursing home residents in the Gulf Coast evacuated before a recent hurricane, 30- and 90-day mortality and hospitalization rates were higher compared with rates during non-hurricane control years [10]. 

### 3.6. Mental Health Effects

Experiencing a hurricane or major natural disaster may exacerbate existing mental health conditions or contribute to new mental health and interpersonal problems [70–74]. Particularly in the months immediately following exposure to a natural disaster, increases in levels of post-traumatic stress disorder (PTSD), as well as other mental health problems have been observed [72]. More than a year after Hurricane Katrina, anxiety and mood disorders in the New Orleans metro area were substantially elevated, and mental health conditions were broadly distributed in the population [75]. Serious mental illness was typically accompanied by PTSD, and important predictors of mental health problems were storm-related physical illness or injury, physical adversity, and property loss. In addition, two years after Katrina, the prevalence of self-reported psychological and physical intimate partner violence increased among Mississippi residents affected by the hurricane [76]. Self-reported poor physical and mental health before and after a storm has also been correlated with self-reported poor mental health after the storm [77]. 

The duration of mental health problems may depend on the nature of exposure to the storm and on ongoing stressors related to the storm. One study of mental health conditions after Hurricane Ike in 2008 showed that prevalence of storm-related PTSD decreased within 18 months [72]. However, elevated levels of PTSD and psychological distress among vulnerable populations have also been observed up to five years after a hurricane [83]. Following Hurricane Katrina, researchers have suggested that slow government responses may have exacerbated mental health problems and argued that an efficient emergency response can also help to minimize the mental health impacts of natural disasters [75].

For those who have experienced displacement, short- and long-term mental health effects are the most commonly cited storms-related health outcomes in the literature. Evacuees at the Red Cross Shelter in Austin, TX, USA, following Katrina, were at increased risk of short-term acute stress disorder, while populations who were displaced or who experienced or witnessed traumatic events were at increased risk of long-term mental health effects, including PTSD, depression, anxiety, and suicidal ideation [70, 77, 78, 81, 82, 84, 85]. Women, African-Americans, and those with prior psychiatric history, poor physical health, and weak social networks have been identified as particularly vulnerable [75, 78, 79, 81, 82, 85]. Katrina evacuees living in Houston were also found to be at risk for increased substance use [80, 86]. 

Many studies on mental health conditions were cross-sectional, and pre-storm depression and PTSD levels could not always be ascertained, thereby limiting conclusions that could be drawn from the data about the cause-effect relationship between storms and subsequent mental health outcomes. Nevertheless, preventing the long-term mental health effects following a storm through ongoing mental health surveillance, appropriate intervention, and adaptation strategies should remain a priority. 

### 3.7. Clean-Up and Reconstruction Work Hazards

The clean-up and recovery period after a major storm may also present significant health risks. Many occupational fatalities have occurred during post-storm clean-up and reconstruction. One study found that, at median, occupational deaths occurred 36.5 days after a storm event [7] and were most often associated with clean-up (44%), restorative construction (26%), public utilities restoration (8%), and security/policing (6%). Residents and volunteers trying to clean affected homes could suffer non-fatal injuries (including cuts, wounds, sprains, and strains) and other health risks during the course of removing debris or during minor and major home repairs (for instance, removing wet building materials or wet wall insulation). Indoor dust created during cleaning, exposure to mold, fumes from temporary heating sources, and the use of strong cleaning products can also irritate the eyes, throat, and lungs. 

The delivery of and access to health care and other basic services—as well as efforts to respond in storm- or flood-damaged areas—could be impeded if workers and volunteers have difficulty traveling to and within the areas. Large-scale displacement could increase demands on the transportation infrastructure, and there may be occupational health concerns for municipal workers deployed to address flooding in transportation and communications infrastructure [7].

During recovery, air quality may be negatively affected by dust from the home clean-up, debris movement, emissions from truck traffic, and the use of outdoor temporary boilers and emergency generators. Following Sandy, routine monitoring at rooftop air monitors in New York City showed that the levels of fine particles (PM_2.5_)—the pollutant most likely to be associated with combustion of fuels, dust from streets, and debris—were not significantly elevated compared to levels typical for the season. Similarly, two-week average PM_2.5_ concentrations at street level monitors near storm- impacted areas in the four weeks after the storm showed that levels were typical for the time of year and similar to those elsewhere in the city. Asbestos was not detected in the dust [87]. In early December 2012, supplemental continuous air monitors were placed in flood-impacted neighborhoods with ongoing recovery operations. For the most part, 24-hour average PM concentrations in these locations through the winter tracked with levels at regional monitors elsewhere in the city, although on several days concentrations were somewhat higher near areas with concentrations of temporary generators and boilers or reconstruction activity [88].

## 4. Summary of Neighborhood Vulnerability

Table 1 summarizes examples of sub-populations identified in prior studies as having increased susceptibility to adverse health effects from different exposures resulting from coastal storms. The health impacts and vulnerable subgroups listed depict a range of potential outcomes but are not exhaustive. For the most part, these studies characterize vulnerability to longer term environmental hazards and stressors from the aftermath of coastal storms rather than the risk of injury or death during the impact phase of a storm. Vulnerable sub-populations vary somewhat by study and specific storm hazard. For the most part, however, groups most vulnerable to adverse storms-related outcomes include one or more of the following: older adults; young children; women; those with pre-existing physical, mental health problems, or substance use disorders; those living in low-income households; members of disadvantaged racial/ethnic groups; and those with weak social networks. 

**Figure 2:**
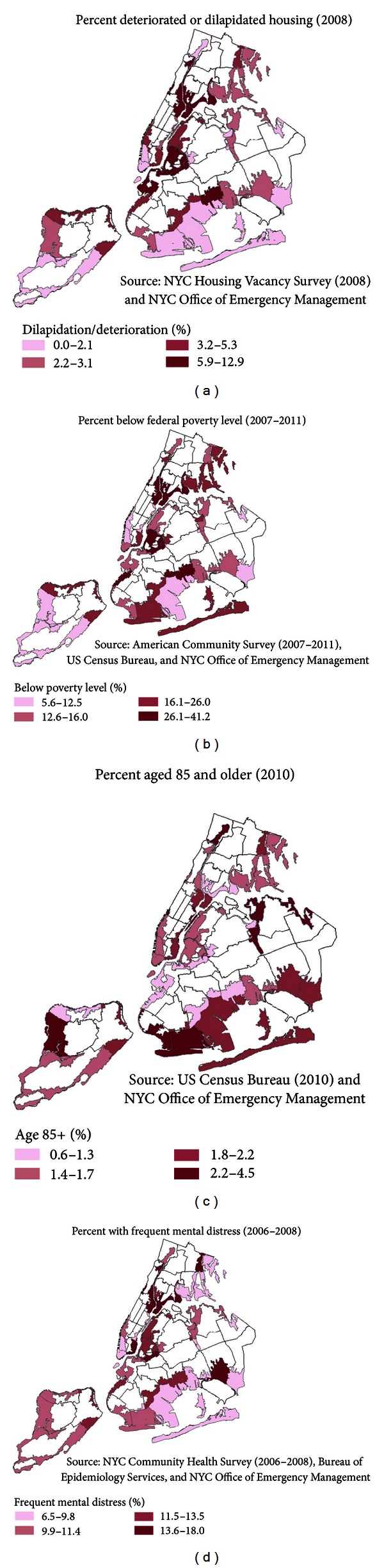
Maps of selected vulnerability indicators within any 2012 NYC hurricane evacuation zone: (a) percent deteriorated or dilapidated housing; (b) percent below federal poverty level; (c) percent aged 85+; and (d) percent with frequent mental distress by UHF neighborhood. Note that prevalence estimates represent the entire UHF neighborhood but are only shown within the evacuation zone.

Based on the literature and data available at the neighborhood level, we identified 18 indicators of vulnerability in the domains of mental health, physical health, socioeconomic status, and housing (Table 2). Examples of indicator maps from each of these domains are shown in Figure 2. Indicators were mapped within any 2012 evacuation zone. 

In NYC, many neighborhoods with high poverty levels also had relatively high levels of other vulnerability indicators. For instance, the percent of people living below the federal poverty level by neighborhood was highly correlated with indicators of population-level prevalence of disability frequent mental distress, social isolation, and poor housing quality, and moderately correlated with prevalence of chronic physical health conditions and lack of health insurance (Table 2). 

## 5. Limitations

The literature review process was not intended to be exhaustive but was rather a means to capture the majority of known health outcomes associated with flooding and coastal storms, and some subjective judgments were used to identify potential health effects for which there are no published studies. However, there may be health effects that are under-represented, not adequately characterized, or not described in the literature. Some potential health risks were added based on unpublished observations following Superstorm Sandy.

Population vulnerability may differ according to geographic region for a variety of reasons, so indicators based on studies from other areas may not adequately describe vulnerability in NYC or other urban areas. Analyses of correlation between indicators and neighborhood poverty do not take variation in survey estimates or potential spatial auto-correlation into account. In addition, we mapped vulnerability indicators within relatively large neighborhood areas. Studies of vulnerability in smaller areas will be important in helping planners and communities better identify and prepare for the potential health impact of storms, especially in assisting in the development and implementation of neighborhood-based interventions. 

It is also important to note that areas outside of evacuation zones are also not without risk. Power outages related to wind damage or damage to utility substations can occur outside of the evacuation zones. Hurricane evacuation zone designations will also likely expand in many places as rising sea levels are factored into the projected storm surge zone. Furthermore, NYC indicators that were available and used for mapping are imperfect proxies for vulnerable subgroups identified in the literature. Vulnerability maps based on these indicators do not represent an exact geospatial representation of vulnerability but rather a proxy measure of vulnerability for research and adaptation purposes [89, 90]. 

## 6. Conclusions 

With predicted warming of the climate, NYC and other coastal cities may be increasingly vulnerable to flooding during hurricanes and other severe storms. These events can have a devastating toll, as witnessed in 2005 with Hurricane Katrina in the Gulf Coast and in 2012 with Superstorm Sandy in the Northeast. A wide range of potential acute and long-term health impacts were identified in the literature, from injury and death resulting from a failure to evacuate safely, physical and mental health problems in displaced populations because of disruption of care or stress, and injury and illness risk during repair and recovery, as well as a range of potential health impacts from exposures in damaged housing and from sheltering in place. Mental health problems were some of the most frequently cited health consequences of major storms. For several dimensions of public health vulnerability to coastal storms, NYC neighborhoods with elevated poverty levels may be at increased risk for lasting impacts.

While adaptation planning must be tailored to the needs of local health jurisdictions, public health protection depends to a great extent on minimizing critical infrastructure vulnerabilities, including enhancing the resilience of power delivery networks (for instance, ensuring that key power infrastructure is located above anticipated high-water lines), buildings, transportation, and health care systems. It is also clear from experiences described in the literature that emergency preparedness for coastal storms will need to include preparation for both short-term and long-term needs of the most vulnerable populations, especially physical and mental health care and access to other essential services. Increasing public health resilience to coastal storms also requires community engagement in climate-readiness strategies and an interdisciplinary approach to adaptation planning.

## Figures and Tables

**Table 1:** Selected impacts and vulnerable subgroups for acute and secondary coastal storm exposure*.

Hazard	Health Impact and References	Vulnerable Subgroups
	*Mortality/injury* CDC/ATSDR Public Health Vulnerability Mapping System; Jonkman et al. (2009) [9]; Brunkard et al. (2008) [6]; and Rygel et al. (2006) [11]	65+ years old Black/African-American Non-English speakers Poor housing quality
Exposure to storm	*Acute myocardial infarction* Gautam et al. (2009) [21]	Unemployed Substance abuse & smoking Uninsured
	*Mental health effects* Picou and Hudson (2010) [78]; Kessler et al. (2008) [70]; Kim et al. (2008) [77]; and Cherry et al. (2010) [71]	Women Black/African-American Low-income Age 40–59 for PTSD Self-report of poor physical health Pre-storm depression Age 45–90 for declines in working memory

	*CO poisoning* Gulati et al. (2009) [31]	Non-English speakers
	*Increase in EMS calls* Rand et al. (2005) [39]; Kile et al. (2005) [32]	Patients who rely on electrically powered medical equipment
Power outage	*Diarrheal illness* Marx et al. (2006) [48]	Meat/seafood consumers
	*Respiratory hospital admissions* Lin et al. (2011) [41]	Age 75+
	*Excess mortality* Anderson and Bell (2012) [35]	Age 65–74

Ingestion (hand to mouth) of contaminated water	*Diarrhea, non-diarrhea GI morbidity, waterborne diseases* Charron et al. (2004) [62]; Curriero et al. (2001)	Immunocompromised Elderly, young children

Living in shelters	*Acute stress disorder* Mills et al. (2007) [79] *Infectious disease*	Prior psychiatric history
	Murray et al. (2009) [61]	Infants

Displacement	*Interruption of chronic disease management* Anderson et al. (2009) [65]; Arrieta et al. (2009) [63]	People with chronic illness such as cancer, hypertension, CV disease, respiratory illness, end stage renal disease, and AIDS Medicaid users People living alone

	*Hip fracture (ages 65+)* Uscher-Pines et al. (2009) [69]	Co-morbid conditions Older age Females Non-African-American race Prior history of hip fracture
Long-term displacement/storm exposure	Increased substance use** Cepeda et al. (2010) [80]	Women High school level education and above
	*Mental health effects* LaJoie et al. (2010) [81]; Abramson et al. (2008)[82]	Women Greater storm exposure Weak social network Increased number of children in single household Fatalistic sentiments and poor self-efficacy

*Selected health impacts and vulnerable subgroups lists depict range of outcomes but are not exhaustive.

**Among low-income, African-American substance users evacuated from New Orleans, LA, USA.

**Table 2:** Selected indicators of neighborhood health vulnerability and correlation with neighborhood poverty levels.

Indicator (age group)	Domain	Data source	Year	Correlation with % below FPL (Pearson's *r* coefficient)	*P* value
% Below federal poverty level	Socioeconomic status	ACS	2007–2011	—	—
% 3 or more maintenance deficiencies	Housing	HVS	2008	0.73	<0.0001
% Deteriorated or dilapidated housing	Housing	HVS	2008	0.72	<0.0001
% Reporting illicit drug use (18+)	Mental health	CHS	2004	−0.05	0.7359
% Reporting social isolation (45+)	Mental health	CHS	2007	0.67	<0.0001
% Ever diagnosed with depression (18+)	Mental health	CHS	2007-2008	0.43	0.005
% Frequent mental distress (18+)	Mental health	CHS	2006–2008	0.73	<0.0001
% Ever diagnosed with high BP (18+)	Physical health	CHS	2007-2008	0.44	0.0033
% Ever diagnosed with diabetes (18+)	Physical health	CHS	2007-2008	0.47	0.0015
% Reporting poor physical health (18+)	Physical health	CHS	2005-2006	0.19	0.2161
% Aged 85+	Physical health	Census	2010	−0.59	<0.0001
% Disabled (16+)	Physical health	Census	2000	0.83	<0.0001
% History of hip fracture (65+)	Physical health	SPARCS	2000–2008	0.16	0.3069
% Aged <5	Physical health	Census	2010	0.69	<0.0001
% Living with HIV/AIDS	Physical health	NYC BHIV/AIDS	2010	0.47	0.0019
% Uninsured (18+)	Physical health	CHS	2007-2008	0.71	<0.0001
% Black non-Hispanic	Socioeconomic status	Census	2010	0.30	0.0529
% Who speak English less than well	Socioeconomic status	ACS	2007–2011	0.42	0.0052

Data Sources: NYC Housing Vacancy Survey (HVS), NYC Community Health Survey (CHS), US Census, NYC DOHMH Bureau of HIV/AIDS (BHIV/AIDS) Prevention & Control Annual Surveillance Statistics, New York State Department of Health, Statewide Planning and Research Cooperative System (SPARCS), and American Community Survey (ACS).
